# Dietary Characteristics of Elders with Frailty and with Mild Cognitive Impairment: Cross-Sectional Findings and Implications from the Nutrition and Health Survey in Taiwan 2014–2017

**DOI:** 10.3390/nu14245216

**Published:** 2022-12-07

**Authors:** Szu-Yun Wu, Shu-Chen Lee, Nai-Hua Yeh, Chi-Fen Wang, Shu-Yi Hung, Shin-Jiuan Wu, Wen-Harn Pan

**Affiliations:** 1 Institute of Biomedical Science, Academia Sinica, 128 Sec. 2, Academia Rd. Nankang, Taipei City 115, Taiwan; 2 Department of Food Nutrition, Chung Hwa University Medical Technology, No. 89, Wenhua 1st St., Rende Dist., Tainan City 717, Taiwan; 3 Institute of Population Health Sciences, National Health Research Institutes, No. 35, Keyan Road, Zhunan Town, Miaoli County 350, Taiwan; 4 Department of Food Nutrition and Health Biotechnology, Asia University, No. 500, Lioufeng Rd., Wufeng, Taichung 41354, Taiwan; 5 Department of Biochemical Science & Technology, National Taiwan University, No. 1, Sec. 4, Roosevelt Rd., Taipei City 10617, Taiwan; 6 Institute of Public Health, National Yang Ming Chiao Tung University, No. 155, Sec. 2, Linong St., Beitou Dist., Taipei City 112, Taiwan

**Keywords:** nutrient intake, RDA, frailty, cognitive impairment, Nutrition and Health Survey in Taiwan

## Abstract

It is unclear whether low dietary intake accompanied with multiple nutrient deficiencies or specific nutrient inadequacy is associated with geriatric syndrome. This study aimed to examine the nutrition inadequacy profiles associated with frailty and cognitive impairment (CI). With information from the Nutrition and Health Survey in Taiwan, 2014–2017, sex-specific nutrient intakes and intake per kg of body weight (BW) were estimated from 24-hour recall data for two age groups (65–74 years; ≥75 years) regarding the three frailty and three CI subgroups. Total energy intakes were significantly lower with the severity of both frailty and CI in analysis combining both gender and age groups, and in both the 65-to-74-year-old women or the over-75-year-old women. These trends were observed but not significant in either of the two age groups in men. Significantly lower levels of energy intake have been observed when age, sex, and sampling strata were adjusted. Intake levels of multiple nutrients also decreased with the severity of frailty and CI. A greater number of nutrient inadequacies for the frail and the CI was found in the 65-to-74-year-old group than the over-75-year-old age group. However, most of the associations between micronutrients and the two geriatric syndromes disappeared after energy adjustment. The remaining few did not show consistency across age–sex subgroups. In conclusion, frailty or CI was associated with low amounts of food consumption accompanied by multiple nutrient insufficiencies. Dietary intervention to ensure adequate total energy and multiple nutrient intakes should be trialed in the geriatric population to address both the causal and efficacy issues.

## 1. Introduction

With the rapidly growing proportion of the older population, the world is facing the challenges associated with this transition to aged society. Healthy aging has been recognized as one of the crucial strategies for sustainable development. From a nutritional point of view, healthy aging could be promoted by ensuring sufficient energy and all essential nutrients to elders. However, little is known about the multiple nutrient insufficiency profiles associated with the “unhealthy” aging process.

Aging is accompanied by multiple physiological dysfunctions. Frailty and cognitive impairment (CI) are the two most common components of the geriatric syndromes [1] and they were identified as risk factors for each other in cross-sectional and longitudinal studies [2,3,4]. Many age-related processes leading to frailty may have an influence on brain aging and cognitive function, and CI is sometimes considered to be a component of frailty [5] and named as a cognitive frailty [6]. Although multiple potential mechanisms have been studied concerning Alzheimer pathology, hormones, nutrition, chronic inflammation, cardiovascular risks, mental health, mitochondrial dysfunction, and oxidative stress, etc. [1,2], it is unclear how the two syndromes are interrelated.

Age itself is a major independent risk factor of frailty and CI [1]. While multiple aging-related factors contribute to the development of frailty and CI, nutrition is a potentially modifiable factor since elderly people tend to gradually lose appetite which may reduce dietary intake and jeopardize human health [7]. On the other hand, people who develop geriatric syndrome may enter into a vicious cycle and further reduce their food intake [8]. Although it is hard to tease apart causal agents and the consequences of these complex circles, documenting the energy and nutrient profiles associated with geriatric syndrome may shed light on major nutrition problems and the interrelationship between geriatric components. In this study, we investigated the nutrient insufficiency profiles associated with frailty and with CI by sex and age groups (65-to-74-year-old age group vs. over-75-year-old age group), taking advantage of data collected from the Nutrition and Health Survey in Taiwan (NAHSIT) where the older population is growing rapidly.

## 2. Methods

### 2.1. Study Population and Ethics

The study population is from the NAHSIT, a nationwide population-based survey executed on a regular basis. In brief, the target population was non-institutionalized Taiwanese nationals and a stratified, three-stage, clustered sampling scheme was used. Details of the survey design and data collection have been published elsewhere [9]. The survey protocol was approved by the Institutional Review Board on Biomedical Science Research, Academia Sinica (project AS-IRB01-13067). All participants provided written informed consent prior to the interview.

### 2.2. Dietary Assessment

Participants’ dietary intake of the past 24-hour before the survey visit was assessed by using a 24-hour dietary recall method, inquiring about the consumed dietary items, the quantity of intake, and cooking recipes. Methods of 24-hour recall and nutrient computation were similar to those employed in 2005–2008 by NAHSIT [10]. Data of the total energy and 24 nutrients including three macronutrients, nine vitamins, seven minerals, three groups of fatty acids, dietary fibre, and cholesterol were used for statistical analysis in this study.

For further investigation into the dietary pattern, the consumed dietary items and their nutrient data were further used for estimating the number of servings of the six major food groups, the same as the Daily Food Guide of Taiwan (grains and roots, soybeans and products, aquatic foods, eggs and meat, vegetables, fruit, dairy, and nuts and oils) [11].

### 2.3. Frailty Definition

Like an earlier study published elsewhere [9], the three severity levels of frailty (robust, pre-frail, and frail) were defined using modified Linda Fried criteria with data and cut-off points from NAHSIT, 2014–2017.

### 2.4. Cognitive Function Assessment

The cognitive function was assessed by the Chinese Mini-Mental State Examination (CMMSE) score [12]. According to Tsai et al. (2016) from Taiwan, participants with the score ≥ 28 were classified as normal cognitive function, those with a score = 24–27 were classified as MCI, and those with a score ≤ 23 were classified as CI [13].

### 2.5. Statistical Analysis

The present study used data of those aged 65 years and above (*n* = 1920) from 2014–2017 NAHSIT. Among them, 1681 provided both dietary intake and MMSE scores, and 1186 provided both dietary intake and frailty data. First of all, the associations between geriatric syndromes (frailty and CI) and nutrient intake indicators (total nutrient intake and total nutrient intake per kg of body weight) were compared within each of the four age-sex groups (65–74-year-old men; over-75-year-old men; 65–74-year-old women; over-75-year-old women). All analyses were weighted and adjusted to obtain population-representative estimates by using SUDAAN (version 11.0.1; RTI International, Research Triangle Park, NC, USA) to account for the complex survey design. Within each age–sex group, a trend test was performed with a generalized linear model to examine whether the severity of frailty or CI had an ordered relationship with total energy and multiple nutrient intakes. Age was adjusted in the models for energy and all nutrients, wherever appropriate. Energy-related factors such as physical activity, body weight, and body mass index were not adjusted in the models because these factors have been used to define frailty. Trend tests were also performed on energy adjusted values to check whether associations between macro- or micro-nutrients and the two geriatric syndromes were independent of energy intake level.

Secondly, the percentage of each nutrient intake indicator relative to its recommended dietary allowance (RDA) or adequate intake (AI) were estimated by age–sex–geriatric syndrome groups for graphical presentation. Reference values of RDAs or AIs were from the eighth version of Taiwanese DRIs [14]. As for fat, cholesterol, fatty acids, sodium, and potassium, no reference values were available in Taiwanese DRIs. Hence, available recommendations from the Health Promotion Agency in Taiwan and the American DRIs were applied, for example, 30% of total energy for total fat intake [11], less than 2400 milligrams for the sodium intake [15], 3400 milligrams for men and 2600 milligrams for women for the potassium intake [16], and less than 300 milligrams for the cholesterol intake [17]. As for fatty acids, the percentage of saturated fatty acids (SFA), monounsaturated fatty acids (MUFA), and polyunsaturated fatty acids (PUFA) were estimated with the total fat intake as the denominator.

## 3. Results

**Figure 1:**
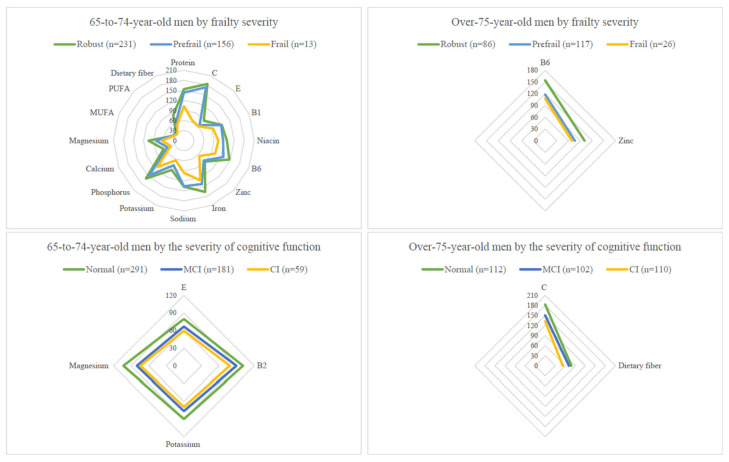
Comparing nutrient intake levels (presented as % RDA) across geriatric syndrome subgroups in men. Top: Total energy and nutrients intakes (presented as % RDA) by three frailty subgroups and by two age groups in men. Bottom: Total energy and nutrients intakes (presented as % RDA) by three CI subgroups and by two age groups in men. The trend test was performed by using the general linear model to test whether the mean of each nutrient variable has an ordered relationship across either the three frailty subgroups or three CI subgroups when age was adjusted.

**Figure 2:**
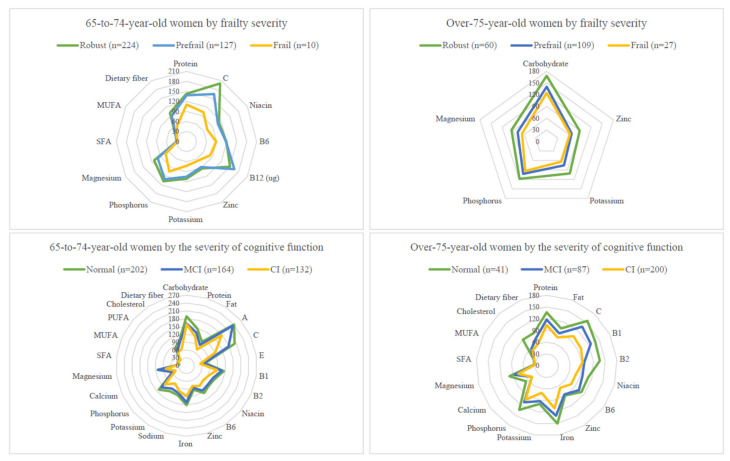
Comparing nutrient intake levels (presented as % RDA) across geriatric syndrome subgroups in women. Top: Total energy and nutrients intakes (presented as % RDA) by three frailty subgroups and by two age groups in women. Bottom: Total energy and nutrients intakes (presented as % RDA) by three CI subgroups and by two age groups in women. The trend test was performed by using the general linear model to test whether the mean of each nutrient variable has an ordered relationship across either the three frailty subgroups or three CI subgroups when age was adjusted.

Age-adjusted nutrient intake means and standard errors by age and by geriatric syndrome groups (frailty or cognitive impairment) in men and women are shown in Supplementary Tables S1–S8. For presentation clarity, only nutrient intake levels with a significant *P* value for trends after age and energy adjustment, expressed as percentage RDAs, by age and geriatric syndrome groups in men and women are shown in Figure 1 and Figure 2.

### 3.1. Total Energy Intake Levels by Sex, Age, and Geriatric Syndrome Subgroups

Total energy intakes were significantly decreased with the severity of frailty (*P* for trend = 0.001) and cognitive impairment (*P* for trend < 0.0001), respectively (Table 1). However, the decreasing trends were significant (or significant at borderline) in the 65-to-74 year-old age group and in over-75-year-old women, but not in men, with respect to the two geriatric syndromes. In addition, we observed significant sex (both *P*s < 0.0001) but no age differences in the total energy intake levels (Table 1).

### 3.2. Nutrient Intake Levels by Sex, Age, and Geriatric Syndrome Subgroups

With respect to either frailty or CI, intake levels of multiple nutrients decreased with the severity. The number of nutrient inadequacies in the 65-to-74-year-old age group outweighed that of the over-75-year-old age group for frailty and CI. Details are described below.

### 3.3. Frailty

Nutrients that were significantly lowered with the advanced stage of frailty in the 65-to-74-year-old men group included vitamin C, dietary fibre, phosphorus, calcium, magnesium (all *P*s for trend <0.0001 for these five nutrients), protein and PUFA (both *P*s for trend <0.001), vitamin B1, niacin, B6, and E, sodium, potassium, iron, zinc, and MUFA (all *P*s for trend <0.05 for these 9 nutrients) (Supplementary Table S1). In the over-75-year-old men group, two nutrients (vitamin B6 and zinc) were significantly lowered in the frail (both *P*s for trend <0.05) compared with the robust (Supplementary Table S1).

In the 65-to-74-year-old women, the nutrients consumed significantly less with advanced frailty levels included niacin and vitamin C (both *P*s for trend <0.0001), magnesium and dietary fibre (both *P*s for trend <0.001), protein, fatty acids (SFA and MUFA), vitamins (B6 and B12), potassium, phosphorus, and zinc (all *P*s for trend <0.05) (Supplementary Table S3). In the over-75-year-old women, frailty was associated with carbohydrate, phosphorus, potassium, magnesium, and zinc (all *P*s for trend <0.05) (Supplementary Table S3).

Many significant trends with the nutrients described above disappeared after adjusting the total energy intake. Nutrients consistently associated in two or more of the four age–sex groups included potassium, magnesium, dietary fibre, and vitamin C (Figure 1 and Figure 2).

### 3.4. Cognitive Impairment

Significant trends of lower intakes of vitamin E and B2, potassium, and magnesium (all *P*s for trend <0.05) were detected with severity of CI in the 65-to-74-year-old men (Supplementary Table S2). In the over-75-year-old men, vitamin C (*P* for trend = 0.028) and dietary fibre (*P* for trend <0.001) had significantly lower trends in those with a higher degree of CI (Supplementary Table S2).

Nutrients consumed significantly less with the advancement of CI stages in the 65-to-74-year-old women included fat, SFA, potassium, magnesium, dietary fibre, niacin, and vitamin B6 (all *P*s for trend <0.0001), carbohydrate, fatty acids (MUFA and PUFA), phosphorus, and vitamin C (all *P*s for trend <0.001) (Supplementary Table S4). Significance levels were slightly less for protein, vitamins (B1, B2, A, and E), sodium, calcium, iron and zinc, and cholesterol (all *P*s for trend <0.05). In the over-75-year-old women, significant ones were protein, fat, fatty acids (SFA and MUFA), dietary fibre, vitamins (B1, B2, niacin, B6, and C), potassium, phosphorus, calcium, magnesium, iron and zinc, and cholesterol (all *P*s for trend <0.05) (Supplementary Table S4).

Again, after adjusting the total energy intake, most significant trends between nutrients and CI disappeared. Nutrients consistently associated in two or more of the four age–sex groups included potassium, dietary fibre, and vitamin C (Figure 1 and Figure 2).

### 3.5. Nutrient Intake Levels Per Kilogram of Body Weight by Sex, Age, and Geriatric Syndrome Subgroups

For either the robust or the normal cognition groups, the intakes of nutrients per kg of body weight were similar in both the 65-to-74-year-old and the over-75-year-old age group (Supplementary Tables S5–S8). However, intakes decreased with more advanced frailty and CI stages. The number of significant nutrients per kg of body weight were pretty similar to those of total nutrient intakes, including findings with or without energy adjustment.

### 3.6. Nutrients Not Reaching 75% DRIs in Elders with Pre-Frailty/Frailty or with MCI/CI

Among those nutrients with significantly decreasing trends across the severity of frailty, zinc, potassium, magnesium, and dietary fibre did not reach 75% DRIs in elders with pre-frailty/frailty in two or more of the age–sex groups.

As for elders with MCI/CI, vitamin E, potassium, calcium, magnesium, and dietary fibre did not reach 75% DRIs in two or more of the age–sex groups.

### 3.7. Dietary Pattern by Geriatric Syndrome Subgroups

**Figure 3:**
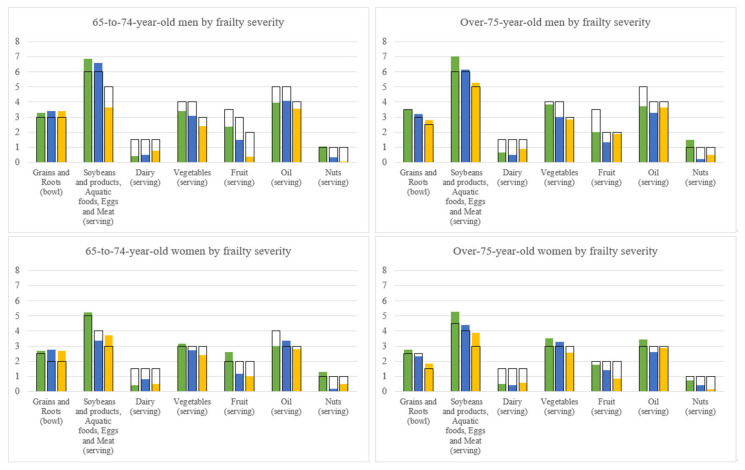
Comparing dietary intake levels of the six food groups with the Daily Food Guide of Taiwan among frailty severity groups by sex and age groups. Top: The dietary intakes of the six food groups by three frailty subgroups and by two age groups in men. Bottom: The dietary intakes of the six food groups by three frailty subgroups and by two age groups in women. The robust is in green, the pre-frail is in blue, and the frail is in yellow. The blank bar (□) indicates the recommended number of servings of the six major food groups by the Daily Food Guide of Taiwan according to the corresponding energy levels.

**Figure 4:**
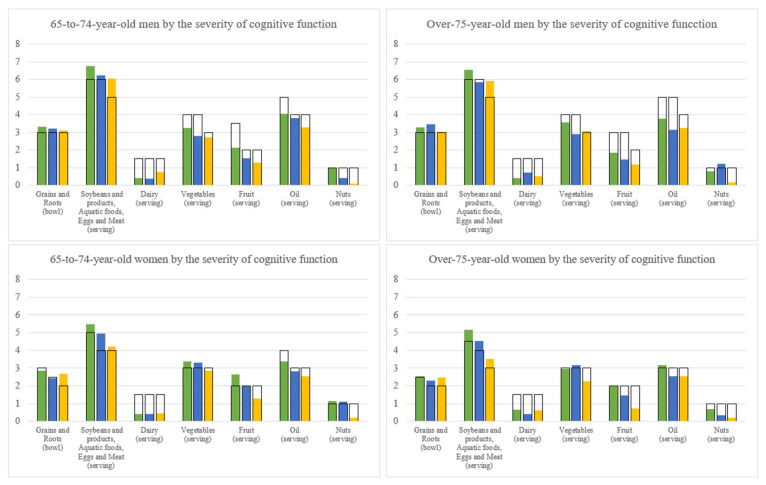
Comparing dietary intake levels of the six food groups with the Daily Food Guide of Taiwan among the severity groups of cognitive function by sex and age groups. Top: The dietary intakes of the six food groups by three cognitive impairment (CI) subgroups and by two age groups in men. Bottom: The dietary intakes of the six food groups by three CI subgroups (the robust is in green, the pre-frail is in blue, and the frail is in yellow) and by two age groups in women. The normal cognitive function is in green, the mild CI is in blue, and the CI is in yellow. The blank bar (□) is the recommended number of servings of the six major food groups by the Daily Food Guide of Taiwan according to the corresponding energy levels.

Figure 3 shows the number of servings of the six major food groups by frailty severity. For comparison purpose, the recommended numbers of serving of the six major food groups suggested by the Daily Food Guide of Taiwan according to the corresponding energy levels were also shown. Figure 4 is for cognitive function.

In the 65-to-74-year-old men and women, when compared with the robust group, the frail group consumed similar servings of grains and roots, but fewer servings in the other four food groups, i.e., protein-rich foods, vegetables, fruit, and nuts and oil. A small amount of dairy was consumed by all three frailty subgroups without consistent patterns. In the over-75-year-old men and women, when compared with the robust group, the frail group consumed less in all major food groups except dairy intake which was modest and similar in the three frailty levels. As for the cognitive function, the CI groups of all 65-to-74-year-old men, 65-to-74-year-old women, over-75-year-old men, and over-75-year-old women consumed fewer servings than the normal groups in all major food groups except dairy.

When compared with the Daily Food Guide, the frail and the CI adults consumed more grains and roots, but mostly fewer servings of dairy, vegetables, fruit, and nuts and oil than the recommendations for their specific energy level. With respect to protein-rich foods (soybeans and products, fish, eggs, or meat), all sex and age groups with geriatric syndromes, except for the 65-to-74-year-old frail men, consumed more than the recommended servings given their energy intake level.

## 4. Discussion

Using data from 2014–2017 NAHSIT, we found that the more severe the frailty or CI level, the lower the intakes of foods, energy as well as multiple nutrients, including macronutrients, multiple vitamins, and minerals. Findings on energy intake and nutrient intake per kg of body weight are not much different from those of the total intake. Most of the significant associations between nutrients and frailty/CI disappeared after energy adjustment. Apparently, this is due to significant correlations between the intakes of these nutrients with total energy. A few nutrients, such as magnesium, potassium, vitamin C, and dietary fibre, remained significantly associated in some but not all age–sex subgroups, indicating a smaller intake of plant foods beyond that of total intakes. However, this did not appear in a consistent fashion in all sex–age groups. The associations between the decreased overall food intake and lower levels of multiple nutrients in those with geriatric syndromes point to the importance of examining the nutrient adequacy of these elders to ensure total wellbeing.

In the vicious cycle diagram of frailty development provided by Fried et al., they proposed that the gradual reduction of food intakes may play a role in the pathogenesis of the physical frailty phenotype [8]. Due to the cross-sectional nature of our analysis, we could not conclude that it is the lower intake of foods which predispose the risk of frailty or MCI, although several prospective studies suggest so. The following studies have reported the significant association between low energy intake and incident frailty to the best of author’s knowledge. An Italian study (sample size = 802, age ≥ 65 years) reported the ORs (95% confidence interval) of total energy intake less than 21 Kcal per kg of body weight (the lowest quintile of the distribution of energy) for frailty was 1.24 (1.02–1.5) [18]. Another British study which included 76 migrant women (age ≥ 60 years) reported the ORs (95% confidence interval) of less than 13 Kcal per kg of body weight (the lowest quintile of the distribution of energy) for frailty was 11.71 (2.36–57.97) [19]. In addition, a Rotterdam study demonstrated that every increase of 100 Kcal in energy intake, but not protein specifically, is associated with 5% lowered ORs of frailty (95% confidence interval, 0.93 to 0.97) [20]. Another Japanese study reported a significantly reversed J-shaped association between the energy intake calibrated with doubly labelled water and the prevalence of physical frailty among community-dwelling older adults [21]. The lowest prevalence of frailty was found to be at approximately 40 Kcal/day/kg ideal body weight. A systematic review included 13 intervention studies and concluded that protein–energy supplementation tended to be effective only in malnourished older adults, and the effects of micronutrient supplementation on frailty were inconclusive [22]. Another meta-analysis of 12 cross-sectional and 5 longitudinal studies found that protein intake, whether absolute, adjusted, or relative to total energy intake, was not significantly associated with frailty in older adults [23]. These results suggest insufficient energy intake might cause lower protein intake which in turn increases the risk of frailty.

For cognition impairment, a recent published meta-analysis of seven studies (total sample size = 808) on Alzheimer’s disease indicates no difference in energy intake between the patients and controls [24]. A recent review article suggested that no single food or nutrient could prevent dementia or cognitive decline, although some of them have shown potential neuroprotective functions [25]. Our cross-sectional study showed low intakes of total energy in MCI elders. We recognize the importance to carry out more prospective investigations.

Findings on lower incidence of frailty in elders with low intake of total energy or foods do not negate the importance of dietary quality. Several previous studies have investigated dietary patterns and frailty and/or cognitive decline [26,27,28,29,30]. The majority have suggested that adhering to a Mediterranean dietary pattern is protective against frailty and dementia [26,27,30,31]. With respect to Asians, including Taiwanese, dietary patterns with more phytonutrient-rich plant foods (vegetable, fruit, nuts, and whole grain), protein-rich foods (dairy, poultry, eggs, soya, and seafood), and tea have been found to be associated with a lower risk of frailty as well as cognitive performance impairment [28,32,33,34]. Our previous intervention studies have demonstrated that a diet fulfilling the Daily Food Guide of Taiwan, i.e., satisfying individualized caloric level and protein requirements, could significantly regress frailty stage and geriatric depression score [35,36]. It is worth noting that the beneficial effect of such a diet may be enlarged when nutrition intervention is combined with physical activity modules [37]. These results of dietary pattern and intervention studies demonstrate the synergistic effects of multiple nutrients with a wide range of physiological functions for preventing frailty and cognitive decline.

With respect to nutrients of low intakes, we found that zinc, potassium, magnesium, and dietary fibre did not reach 75% DRIs in elders with pre-frailty/frailty in two or more of the age– sex groups and, for elders with MCI/CI, vitamin E, potassium, calcium, magnesium, and dietary fibre did not. This may indicate that the plant foods such as vegetables, fruits, and nuts should be worthy of attention, although a causal relationship is not known. One recently reported meta-analysis, including 10 cohorts and 4 cross-sectional studies, showed an inverse association between increment in fruit and vegetables intake and lowered risk of frailty [37]. Frailty risk was reduced by 14% by every serving (200 g) of fruit and vegetables per day with up to 3.5 servings. However, the protection was not observed when fruits and vegetables were separately analyzed. Another meta-analysis including 16 studies with a cross-sectional, cohort, and case-control study design also showed the protective effect of consuming fruit and vegetables alone and together on cognitive disorders in older adults [38]. These results revealed the importance of increasing nutrient density by consuming more fruits and vegetables in the elderly. Food group analysis showed that both frailty or CI elders tended to eat relatively more rice but less amounts of other food groups, again supporting the importance of increasing nutrient density from eating nutrient dense foods. An effective approach for preventing geriatric syndromes would be for elders to consume, on the basis of individualized caloric levels, palatable local gourmet made with properly balanced whole foods.

Several limitations of this study should be noted. First, NAHSIT is a cross-sectional study, which cannot conclude on causality. Second, as compared with the sample size of robust and pre-frail groups, the sample size of the frail group was relatively small and thus may limit the statistical power. This has also limited us to exclude participants with both frailty and CI when carrying out the analysis of the counterpart. Third, the 24-hour diet recall is highly dependent upon one’s memory, which could have produced some systematic biases and non-differentiable misclassifications.

## 5. Conclusions

In conclusion, we demonstrated that Taiwanese elders with frailty or with CI are likely to consume lower levels of total energy as well as multiple nutrients, and multiple nutrient insufficiencies are likely due to the lower level of food intakes. It is essential to boost up appetite and the quality of foods consumed by these elders to ensure health and total wellbeing, particularly for some nutrients with low percentage DRIs such as zinc, potassium, magnesium, fiber, calcium, and vitamin E. Further intervention research focusing on appetite and dietary means to increase nutrient density is highly warranted.

## Figures and Tables

**Table 1:** Energy intakes by geriatric syndrome groups in all participants and in sex–age subgroups, using 2014–2017 NAHSIT data ^a^.

	*n*	Geriatric Syndrome Groups			*P* Values		
					Sex	Age	Trend ^b^
		*Robust*	*Prefrail*	*Frail*			
All participants	601/509/76	1869 ± 41.34	1693 ± 40.93	1537 ± 81.20	<0.0001	0.103	0.001
Men							
65~74-year-old	231/156/13	2078 ± 49.12	1953 ± 74.79	1761 ± 192.7	-	-	0.114
≥75-year-old	86/117/26	2164 ± 122.7	1888 ± 107.8	1840 ± 179.7	-	-	0.158
							
Women							
65~74-year-old	224/127/10	1697 ± 61.18	1629 ± 62.04	1426 ± 106.1	-	-	0.027
≥75-year-old	60/109/27	1484 ± 66.16	1313 ± 66.19	1246 ± 117.4	-	-	0.085
							
		*Normal*	*MCI*	*CI*			
All participants	646/534/501	1851 ± 40.22	1695 ± 38.64	1553 ± 44.45	<0.0001	0.217	<0.0001
Men							
65~74-year-old	291/181/59	2045 ± 43.04	1872 ± 65.68	1829 ± 171.9	-	-	0.254
≥75-year-old	112/102/110	1945 ± 88.32	2045 ± 135.4	1770 ± 125.4	-	-	0.267
							
Women							
65~74-year-old	202/164/132	1761 ± 60.54	1530 ± 72.96	1409 ± 46.63	-	-	<0.0001
≥75-year-old	41/87/200	1594 ± 127.3	1383 ± 72.93	1314 ± 49.97	-	-	0.043

^a^ Data are sex and age groups adjusted Mean ± SEM for all participants, and age-adjusted Mean ± SEM for all the subgroups. CI: cognitive impairment; MCI: mild cognitive impairment. ^b^ The trend test was performed by using the general linear model to test whether the mean energy intake has an ordered relationship across the severity of frailty or CI groups after adjusting for sex and age groups for all participants, or adjusting for age for all the subgroups.

## Data Availability

Data of Nutrition and Health Survey from Taiwan described in the manuscript belong to HPA, which can be accessed in data center with the permission of HPA.

## Supplementary Materials

The following supporting information can be downloaded at: https://www.mdpi.com/article/10.3390/nu14245216/s1, Table S1: Total energy and nutrients intakes for the older men by frailty levels in 2014–2017 NAHSIT. Table S2: Total energy and nutrients intakes for the older men by stages of cognitive ability in 2014–2017 NAHSIT. Table S3: Total energy and nutrients intakes for the older women by frailty levels in 2014–2017 NAHSIT. Table S4: Total energy and nutrients intakes for the older women by stages of cognitive ability in 2014–2017 NAHSIT. Table S5: Total energy and nutrients intakes per kilogram of body weight for the older men by frailty levels in 2014–2017 NAHSIT. Table S6: Total energy and nutrients intakes per kilogram of body weight for the older men by stages of cognitive ability in 2014–2017 NAHSIT. Table S7: Total energy and nutrients intakes per kilogram of body weight for the older women by frailty levels in 2014–2017 NAHSIT. Table S8: Total energy and nutrients intakes per kilogram of body weight for the older women by stages of cognitive ability in 2014–2017 NAHSIT.
