# Supplementary material for: Dietary Characteristics of Elders with Frailty and with Mild Cognitive Impairment: Cross-Sectional Findings and Implications from the Nutrition and Health Survey in Taiwan 2014–2017
Source: Nutrients. 2022 Dec 7;14(24):5216. doi: 10.3390/nu14245216 (PMC9782975; doi:10.3390/nu14245216)
Supplement: Supplementary file 1 [file nutrients-14-05216-s001.zip › nutrients-2027649-supplementary.pdf]

Table S1: Total energy and nutrients intakes for the older men by frailty levels in 2014-2017 NAHSIT [a]

	65-74 year-old men					> 74 year-old men				
	Robust	Prefrail	Frail	*P* for trend [b]		Robust	Prefrail	Frail	*P* for trend [b]	
	(n=231)	(n=156)	(n=13)	*Model 1*	*Model 2*	(n=86)	(n=117)	(n=26)	*Model 1*	*Model 2*
Energy (Kcal)	2078.06±49.12	1952.8±74.79	1760.89±192.73	0.114	0.974	2163.77±122.69	1888.42±107.84	1840.41±179.71	0.158	0.056
	(107.4)	(100.9)	(91.7)			(115.5)	(99.1)	(91.7)		
Carbohydrate (g)	274.18±6.61	261.32±10.14	257.07±28.98	0.567	0.232	280.6±9.21	252.27±13.12	244.69±21.92	0.179	0.537
	(210.8)	(201.2)	(198.8)			(219)	(193.5)	(178.2)		
Protein (g)	86.92±2.78	80.92±3.45	58.3±6.53	<.001	0.133	92.27±6.25	76.71±5.45	74.08±8.83	0.113	0.294
	(153.8)	(143.5)	(103.1)			(155.3)	(127.6)	(118.7)		
Fat (g)	69.07±2.79	63.61±3.91	57.24±11.35	0.311	0.764	71.9±8.36	62.3±5.66	63.39±8.22	0.477	0.117
	(107.2)	(98.5)	(88.7)			(115.1)	(98.1)	(95.1)		
Vitamin A (μg RE)	1179.69±104.69	1019.94±128.2	989.07±220.62	0.446	0.963	1004.54±118.64	980.31±121.56	1278.28±301.22	0.424	0.323
	(194.7)	(172.1)	(180.9)			(170.2)	(162.9)	(204.4)		
Vitamin C (mg)	184.52±9.76	171.41±17.69	56.45±18.19	<.0001	<.0001	197.73±22.89	167.64±17.9	128.34±34.98	0.063	0.083
	(183.5)	(172.6)	(65.4)			(198)	(167.6)	(127.5)		
Vitamin D (μg)	7.1±0.65	7.19±0.74	7.07±3.64	0.994	0.789	10.81±1.56	7.94±1.44	9.07±4.29	0.697	0.969
	(70.5)	(72.4)	(74.6)			(104.1)	(80.1)	(103.3)		
Vitamin E (mg α-TE)	10.16±0.49	7.87±0.61	6.68±1.55	0.036	0.331	10.49±0.79	7.83±0.68	8.69±1.39	0.271	0.473
	(84.1)	(66.3)	(60.6)			(86.1)	(65.5)	(76.4)		
Vitamin B1 (mg)	1.48±0.06	1.43±0.08	1.02±0.17	0.014	0.310	1.53±0.1	1.42±0.12	1.42±0.21	0.616	0.438
	(122.4)	(120.4)	(94.2)			(131)	(117.8)	(107.3)		
Vitamin B2 (mg)	1.35±0.06	1.22±0.08	1.14±0.15	0.211	0.888	1.38±0.1	1.15±0.09	1.26±0.26	0.670	0.949
	(103.1)	(94.4)	(93)			(106.4)	(88.3)	(95.6)		
Niacin (mg)	20.52±1.03	18.73±1.24	16.36±1.82	0.045	0.758	19.31±1.35	18.73±1.4	14.77±1.94	0.060	0.122
	(128.2)	(117.1)	(103)			(121.8)	(116.8)	(88.7)		
Vitamin B6 (mg)	2.36±0.1	2.03±0.15	1.53±0.36	0.024	0.285	2.48±0.14	1.89±0.13	1.7±0.23	0.008	0.007
	(147)	(127.6)	(101)			(154.4)	(118.1)	(107.7)		

Vitamin B12 (μg)	5.4±0.71 (224.4)	5.3±0.9 (221.4)	5.53±1.87 (233.7)	0.944	0.569	4.78±0.7 (202.7)	3.67±0.57 (152.2)	3±0.54 (113.4)	0.058	0.245
Zinc (mg)	13.33±0.47 (88.7)	12.55±0.64 (83.8)	9.63±1 (65.2)	0.001	0.315	14.91±1.23 (100.7)	11.4±0.63 (75.8)	10.82±1.41 (68.1)	0.036	0.015
Iron (mg)	16.63±0.72 (165.8)	13.97±0.92 (140.2)	12.43±1.22 (128.5)	0.004	0.170	17.39±1.65 (177.1)	13.65±0.84 (135.9)	15.49±2.75 (145)	0.556	0.841
Sodium (mg)	3288.57±129.52 (137.5)	3281.1±247.11 (136.2)	2389.45±363.25 (95.8)	0.013	0.288	3668.86±345.53 (153.2)	3082.91±214.4 (128.4)	2787.23±584.5 (115.1)	0.200	0.420
Potassium (mg)	3231.47±117.89 (94.6)	2692.77±158.8 (79.7)	2026.57±325.59 (63.6)	0.001	0.032	3351.44±223.41 (98.7)	2562.15±173.46 (75.3)	2692.59±562.29 (78.8)	0.314	0.538
Phosphorus (mg)	1284.93±42 (160.3)	1170.26±53.83 (146.6)	867.95±77.2 (111.2)	<.0001	0.059	1427.69±168.14 (179.2)	1107.64±70.08 (138.3)	1104.25±144.56 (135.7)	0.167	0.380
Calcium (mg)	655.4±40.61 (65.2)	520.61±44.71 (52.5)	392.25±36.49 (42.2)	<.0001	0.016	671.53±60.84 (67.3)	557.23±52.55 (55.7)	662.51±190.97 (65.9)	0.966	0.627
Magnesium (mg)	377.67±14.11 (105.6)	295.92±18.09 (83.3)	221.43±18.41 (65.2)	<.0001	0.003	430.08±58.48 (124.4)	293.62±16.19 (83.6)	339.34±65.59 (92.3)	0.309	0.665
SFA (mg)	19590.99±843.81 (28.6)	19867.83±1130.71 (31.9)	23459.64±7400.67 (37.3)	0.605	0.166	20711.52±2432.81 (29.3)	20682±1856.32 (33.6)	19667.18±2387.86 (32.1)	0.759	0.025
MUFA (mg)	25650.66±1171.31 (37)	23969.69±1469.13 (37.1)	18628.55±2681.15 (34.7)	0.019	0.422	27148.46±3572.94 (37)	23975.75±2327.48 (37.4)	24779.39±3694.37 (38.1)	0.641	0.090
PUFA (mg)	23004.89±1097.35 (33.2)	19342.99±1567.63 (30.2)	14876.38±1970.33 (27.5)	<.001	0.175	23620.16±2955.37 (33.1)	17216.94±1689.6 (28.2)	18368.23±3223.01 (28.9)	0.240	0.948
Cholesterol (mg)	264.81±13.38 (88.6)	291.72±18.35 (96.9)	179.26±46.37 (57.4)	0.083	0.356	252.64±22.31 (84.8)	247.37±23.39 (82.4)	215.4±38.23 (69.9)	0.419	0.879
Dietary fiber (g)	22.41±0.89 (82.6)	16.28±1.12 (60.8)	12.99±1.92 (51.5)	<.0001	<.001	23.1±2.11 (85.9)	16.78±1.41 (62.1)	18.74±2.98 (68.3)	0.252	0.483

[a] Data are age-adjusted Mean±SEM (% RDA). The RDAs are the current dietary reference intakes (DRIs, the 8th edition) established for Taiwanese people.

[b] The trend test was performed by using the general linear model to test whether the mean of variable has an ordered relationship across frailty level groups after adjusting for age (Model 1) or for age and energy intake (Model 2).

Table S2: Total energy and nutrients intakes for the older men by stages of cognitive ability in 2014-2017 NAHSIT [a]

	65-74 year-old men					> 74 year-old men				
	Normal cognition (n=291)	Mild cognitive impairment (n=181)	Cognitive impairment (n=59)	*P* for trend [b]		Normal cognition (n=112)	Mild cognitive impairment (n=102)	Cognitive impairment (n=110)	*P* for trend [b]	
				Model 1	*Model 2*				*Model 1*	*Model 2*
Energy (Kcal)	2044.68±43.04 (105.9)	1872.27±65.68 (96.6)	1828.5±171.93 (93.8)	0.254	0.350	1945.18±88.32 (103.1)	2045.03±135.37 (107.2)	1769.64±125.38 (92.8)	0.267	0.910
Carbohydrate (g)	271.15±7.05 (209.2)	250.19±9.59 (191.8)	248.7±17.74 (189.4)	0.249	0.902	260.18±10.24 (201.7)	270.67±13.32 (207.3)	236.61±12.68 (181.3)	0.151	0.329
Protein (g)	85.15±2.66 (151.8)	76.62±2.63 (135.1)	76.37±9.85 (133.4)	0.410	0.910	82.12±4.57 (137.5)	82±5.58 (136.3)	72.47±6.85 (120.5)	0.256	0.600
Fat (g)	67.61±2.3 (105.1)	61.23±3.4 (94.7)	56.78±8.86 (87)	0.274	0.643	62.82±5 (99.5)	67.57±8.83 (106.5)	58.33±6.28 (92)	0.596	0.182
Vitamin A (μg RE)	1166.27±101.18 (191.5)	983.89±113.88 (166.7)	758.93±204.97 (135)	0.091	0.126	1018.43±102.72 (168.9)	1018.03±94.19 (170.1)	944.24±145.83 (157.7)	0.657	0.872
Vitamin C (mg)	171.11±9.69 (170.4)	151.47±10.9 (152.1)	142.48±26.24 (144.6)	0.313	0.614	181.85±15.51 (182.7)	150.83±16.94 (150.3)	133.03±15.29 (132.7)	0.028	0.036
Vitamin D (μg)	7.36±0.61 (72.6)	6.8±0.67 (68.9)	5.86±1.76 (61.3)	0.439	0.647	10.06±1.63 (98.9)	8.23±1.2 (83.3)	8.19±1.63 (82.6)	0.433	0.635
Vitamin E (mg α-TE)	9.6±0.35 (79.7)	7.97±0.48 (66.8)	7.02±0.98 (59.7)	0.021	0.011	8.77±0.64 (72.5)	9.07±0.77 (75.9)	8.52±0.9 (71.2)	0.819	0.696
Vitamin B1 (mg)	1.46±0.05 (121.2)	1.29±0.06 (108)	1.23±0.16 (105.5)	0.211	0.389	1.44±0.08 (120.7)	1.4±0.08 (115.9)	1.36±0.17 (112.6)	0.658	0.801
Vitamin B2 (mg)	1.32±0.07 (100.8)	1.15±0.05 (89.5)	0.98±0.14 (78.2)	0.027	0.011	1.17±0.06 (89.5)	1.28±0.08 (98.4)	1.11±0.11 (85.3)	0.614	0.746
Niacin (mg)	19.83±0.9 (124.6)	17.09±1.01 (106.2)	21.11±3.99 (129.9)	0.755	0.393	19.2±1.25 (120.6)	17.01±1.46 (106)	15.83±1.82 (98.6)	0.144	0.189
Vitamin B6 (mg)	2.28±0.08	1.97±0.11	1.78±0.27	0.082	0.161	2.11±0.15	1.98±0.08	1.86±0.18	0.316	0.486

	(141.7)	(123.6)	(112.6)			(131.9)	(123.6)	(116.4)		
Vitamin B12 (μg)	5.64±0.79	4.18±0.38	3.61±0.71	0.066	0.207	4.34±0.56	3.92±0.48	3.53±0.65	0.357	0.593
	(234.6)	(174.6)	(151.8)			(182.9)	(162)	(146.1)		
Zinc (mg)	12.93±0.42	11.84±0.6	11.93±1.22	0.452	0.450	12.07±0.57	13.25±1.06	10.86±0.86	0.238	0.688
	(86.5)	(78.6)	(78.7)			(81.4)	(87.8)	(72)		
Iron (mg)	15.81±0.61	13.52±0.63	13.61±1.99	0.306	0.651	15.08±0.76	15.51±1.37	13.36±1.09	0.182	0.630
	(157.7)	(135.6)	(137.3)			(152.2)	(154.2)	(133)		
Sodium (mg)	3169.46±130.9	3410.61±202.59	2611.1±332.99	0.089	0.185	2977.12±250.65	3111.21±281.45	2987.22±261.32	0.976	0.322
	(133.6)	(140.6)	(104.2)			(124.3)	(129.5)	(124.3)		
Potassium (mg)	3073.49±98.39	2583.9±126.71	2312.1±348.14	0.047	0.016	2923.83±156.27	2729.76±161.19	2572.11±271.67	0.279	0.506
	(89.9)	(76.4)	(69.4)			(86.2)	(80.2)	(75.6)		
Phosphorus (mg)	1245.55±39.03	1094.01±39.2	1081.3±124.46	0.227	0.443	1162.3±53.52	1348.24±139.98	1047.08±89.96	0.289	0.926
	(155.6)	(136.8)	(135.3)			(146.1)	(168.1)	(130.5)		
Calcium (mg)	622.15±33.61	506.05±32.92	520.38±93.66	0.315	0.551	565.2±37.02	653.71±50.37	546.02±56.65	0.771	0.603
	(61.8)	(51)	(53.3)			(56.3)	(65.5)	(54.7)		
Magnesium (mg)	369.23±13.87	284.78±12.73	260±34.11	0.006	<.001	332.88±14.82	380.39±52.57	285.16±26.11	0.104	0.642
	(103.1)	(80.2)	(74)			(96.2)	(108.1)	(81)		
SFA (mg)	19995.49±685.75	18842.82±1198.72	16914±2553	0.278	0.642	19456.94±1425.06	20160.4±2582.4	17809.21±2316.03	0.566	0.570
	(29.8)	(30.8)	(30.1)			(31.5)	(30.4)	(31)		
MUFA (mg)	25294.29±960.03	23038.95±1196.66	21424±3654.4	0.345	0.809	23671.54±2165.49	26187.8±3791.7	21799.53±2676.89	0.605	0.299
	(37)	(37.7)	(37.5)			(37.1)	(37.9)	(36.9)		
PUFA (mg)	21603.16±951.42	18867.49±1334.4	18079.8±2742	0.260	0.697	19274.73±1613.76	20851.2±3052.6	18262.92±1905.55	0.689	0.249
	(32.1)	(30.6)	(31.7)			(30.7)	(31)	(31.2)		
Cholesterol (mg)	265.37±14.49	294.58±22.26	237.22±31.19	0.369	0.834	237.63±20.52	241.68±17.74	233.13±29.66	0.907	0.551
	(88.9)	(97.8)	(77.8)			(79.2)	(80.5)	(77.7)		
Dietary fiber (g)	20.73±0.84	16.02±0.88	16.33±2.6	0.123	0.261	21.06±1.19	19.15±1.9	14.2±1.27	<.001	<.001
	(76.5)	(59.6)	(61.2)			(78.2)	(70.8)	(52.5)		

[a] Data are age-adjusted Mean±SEM (% RDA). The RDAs are the current dietary reference intakes (DRIs, the 8th edition) established for Taiwanese people.

[b] The trend test was performed by using the general linear model to test whether the mean of variable has an ordered relationship across frailty level groups after adjusting for age (Model 1) or for age and energy

intake (Model 2).

Table S3: Total energy and nutrients intakes for the older women by frailty levels in 2014-2017 NAHSIT [a]

	65-74 year-old women					> 74 year-old women				
	Robust	Prefrail	Frail	*P* for trend [b]		Robust	Prefrail	Frail	*P* for trend [b]	
	(n=224)	(n=127)	(n=10)	*Model 1*	*Model 2*	(n=60)	(n=109)	(n=27)	*Model 1*	*Model 2*
Energy (Kcal)	1697.46±61.18	1629.34±62.04	1425.63±106.08	0.027	0.954	1483.5±66.16	1312.88±66.19	1245.79±117.38	0.085	0.872
	(108)	(103.8)	(91.6)			(99.9)	(87.5)	(81.6)		
Carbohydrate (g)	236.92±7.06	227.44±8.75	214.75±11.07	0.086	0.325	215.94±8.72	182.93±8.58	166.97±16.23	0.011	0.023
	(182.5)	(174.5)	(161.6)			(168.6)	(140.7)	(124.7)		
Protein (g)	71.57±2.94	69.29±4.3	55.57±7.46	0.046	0.319	60.74±3.44	52.41±3.5	50.91±4.44	0.069	0.916
	(143.2)	(138.5)	(110.7)			(123)	(104.8)	(99.5)		
Fat (g)	54.11±3.01	50.52±3.38	40.95±6.27	0.059	0.704	42.62±3.43	42.61±3.48	43.08±6.36	0.950	0.006
	(103.2)	(96.6)	(80.8)			(85.4)	(85.2)	(86)		
Vitamin A (μg RE)	1110.77±93.27	1229.68±212.12	1672.15±1111.15	0.616	0.508	1625.58±547.56	820.1±125.87	893.29±184.01	0.180	0.255
	(222)	(246.1)	(335.9)			(344.3)	(163.5)	(149.8)		
Vitamin C (mg)	200.14±14.62	165.03±14.78	108.65±14.11	<.0001	0.001	157.03±21.31	112.64±12.67	110.11±28.32	0.212	0.256
	(200.7)	(164.2)	(101.3)			(159)	(112.6)	(107.2)		
Vitamin D (μg)	5.85±0.56	6.91±0.87	4.27±1.04	0.227	0.622	5.03±1.02	5.21±0.71	4.73±1.23	0.845	0.642
	(58.2)	(69.5)	(45.3)			(52)	(52.1)	(44.8)		
Vitamin E (mg α-TE)	8.5±0.51	8.19±0.58	8.72±1.62	0.902	0.321	6.81±0.88	6.67±0.56	6.27±0.95	0.673	0.432
	(70.7)	(68.4)	(74)			(58.2)	(55.6)	(50.2)		
Vitamin B1 (mg)	1.28±0.06	1.24±0.08	1.19±0.17	0.621	0.606	1.07±0.09	0.95±0.08	0.98±0.17	0.629	0.571
	(142.6)	(137.8)	(129.5)			(119.7)	(106.1)	(107.6)		
Vitamin B2 (mg)	1.15±0.06	1.17±0.09	1.24±0.25	0.696	0.284	1.15±0.09	0.89±0.07	0.99±0.13	0.284	0.969
	(114.8)	(117.5)	(125.8)			(115.8)	(89)	(97.5)		
Niacin (mg)	15.75±0.61	15.11±0.8	9.95±1	<.0001	0.001	14.4±1.25	11.15±0.91	12.24±1.53	0.334	0.915
	(112.4)	(108)	(71.6)			(102.6)	(79.6)	(87.8)		
Vitamin B6 (mg)	1.91±0.09	1.89±0.13	1.42±0.17	0.009	0.408	1.68±0.14	1.35±0.07	1.31±0.15	0.062	0.344

	(119.1)	(118)	(88.8)			(106.7)	(84.3)	(79.2)		
Vitamin B12 (μg)	3.63±0.33	3.93±0.44	1.68±0.4	0.001	0.016	4.36±1	3.59±0.77	6.36±3.73	0.621	0.543
	(150.3)	(165.2)	(81.9)			(193.1)	(149.3)	(247.5)		
Zinc (mg)	11.17±0.49	10.56±0.52	8±1.24	0.020	0.133	10.41±0.92	8.09±0.46	7.96±0.76	0.028	0.199
	(93.1)	(88)	(66.6)			(89.1)	(67.3)	(62.8)		
Iron (mg)	14.55±0.73	14.49±1.44	11.84±1.67	0.140	0.848	13.44±0.74	11.02±0.75	11.22±1.33	0.153	0.657
	(145.4)	(145.1)	(119.6)			(139.8)	(110.1)	(104.3)		
Sodium (mg)	2668.7±151.7	2829.44±176.11	2278.65±262.14	0.190	1.000	2223.44±153.65	2488.19±262.51	2121.46±247.93	0.739	0.421
	(111.4)	(117.6)	(92.5)			(94)	(103.6)	(86.3)		
Potassium (mg)	2891.03±131.49	2734.68±201.87	1879.99±318.67	0.004	0.116	2591.76±149.49	1964.94±128.38	1718.82±237.82	0.003	0.005
	(111.2)	(105.2)	(72.8)			(101.2)	(75.5)	(63.9)		
Phosphorus (mg)	1096.87±47.31	1042.49±57.85	827.36±62.68	0.001	0.099	935.7±42.3	819.64±52.07	761.21±75.54	0.042	0.499
	(137.1)	(130.3)	(103.5)			(118.2)	(102.4)	(93.3)		
Calcium (mg)	593.53±45.48	603.9±76.97	501.51±108.51	0.438	0.828	588.71±40.95	461.87±44.43	434.62±78.85	0.085	0.297
	(59.4)	(60.4)	(50)			(60.7)	(46.1)	(40.7)		
Magnesium (mg)	344.06±16.17	305.9±21.77	224.27±25.45	<.001	0.031	277.68±11.91	234.6±15.95	209.34±25.54	0.024	0.114
	(112.1)	(99.4)	(71.9)			(94.8)	(78.1)	(66.4)		
SFA (mg)	15581.82±1011.76	14953.82±969.56	11007.63±1736.63	0.025	0.370	12682.14±1027.16	13007.56±1113.73	13148.6±2478.6	0.863	0.046
	(29.1)	(30.1)	(26.9)			(29.9)	(30.4)	(30.6)		
MUFA (mg)	20770.1±1209.46	18533.51±1141.02	13677.85±2294.25	0.007	0.042	15781.49±1232.9	15592.15±1442.76	15675.46±2406.56	0.969	0.013
	(38)	(36.5)	(34.1)			(37.3)	(35.7)	(35.7)		
PUFA (mg)	17285.58±1085.02	16675.59±1548.92	15621.37±2931.9	0.597	0.313	13829.31±1358.5	13553.26±1076.44	13958.6±1876.03	0.957	0.040
	(32)	(32.6)	(37.1)			(31.9)	(32.6)	(32.8)		
Cholesterol (mg)	207.85±17.53	258.57±26.24	161.39±62.77	0.470	0.965	193.56±33.81	162.93±22.06	202.05±29.48	0.836	0.216
	(69.3)	(86.2)	(53.5)			(65.6)	(54.3)	(65.8)		
Dietary fiber (g)	21.53±0.87	19.26±1.36	12.08±2.28	<.001	0.009	16.49±1	13.43±1.07	13.09±2.01	0.196	0.554
	(99.1)	(88.7)	(55.6)			(80.8)	(63.9)	(59)		

[a] Data are age-adjusted Mean±SEM (% RDA). The RDAs are the current dietary reference intakes (DRIs, the 8th edition) established for Taiwanese people.

[b] The trend test was performed by using the general linear model to test whether the mean of variable has an ordered relationship across frailty level groups after adjusting for age (Model 1) or for age and energy

intake (Model 2).

Table S4: Total energy and nutrients intakes for the older women by stages of cognitive ability in 2014-2017 NAHSIT [a]

	65-74 year-old women					> 74 year-old women				
	**Normal cognition**	**Mild cognitive impairment**	**Cognitive impairment**	***P* for trend [b]**		**Normal cognition**	**Mild cognitive impairment**	**Cognitive impairment**	***P* for trend [b]**	
	**(n=202)**	**(n=164)**	**(n=132)**	***Model 1***	***Model 2***	**(n=41)**	**(n=87)**	**(n=200)**	***Model 1***	***Model 2***
Energy (Kcal)	1761.58±60.54 (112.2)	1530.65±72.96 (97.8)	1408.65±46.63 (90.5)	<.0001	0.971	1593.77±127.34 (108.6)	1383.28±72.93 (92.5)	1314.19±49.97 (86.9)	0.043	0.973
Carbohydrate (g)	245.94±7.18 (189.4)	210.29±8.74 (161.7)	206.79±7.47 (158.6)	<.001	0.878	212.27±13.38 (168.4)	191.35±8.04 (147.8)	190.11±7.96 (144.7)	0.178	0.387
Protein (g)	73.97±3.15 (147.8)	66.27±4.02 (132.6)	58.26±3.36 (116.8)	0.001	0.748	67.16±3.65 (136.5)	58.74±5.55 (117.7)	52.33±2.76 (104)	0.002	0.705
Fat (g)	57.01±2.83 (108.8)	49.54±3.46 (95)	38.22±2.6 (74.2)	<.0001	0.157	50.4±5.17 (102.2)	44.28±4.98 (88.7)	38.92±2.45 (77.4)	0.041	0.933
Vitamin A (μg RE)	1207.19±118.45 (241.3)	1170.65±136.69 (234.1)	888.15±105.98 (177.9)	0.027	0.572	1763.17±809.52 (373.6)	962.85±82.77 (195.1)	827.76±89.37 (159.1)	0.246	0.339
Vitamin C (mg)	202.25±16.19 (202.3)	176.78±14.84 (176.8)	120.33±10.3 (120.2)	<.001	0.014	153.21±18.01 (154.7)	134.68±13.05 (134.9)	102.51±10.28 (102)	0.013	0.047
Vitamin D (μg)	6.11±0.69 (60.8)	6.38±1.09 (63.8)	5.15±0.76 (52.1)	0.418	0.802	4.09±0.61 (45.2)	6.28±1.03 (63.3)	5.28±0.59 (51.5)	0.175	0.012
Vitamin E (mg α-TE)	8.65±0.45 (72)	8.04±0.52 (67.1)	6.35±0.44 (53.2)	0.001	0.247	7.34±0.92 (63.4)	7.29±0.74 (61)	6.32±0.39 (52)	0.288	0.847
Vitamin B1 (mg)	1.3±0.06 (144.4)	1.23±0.08 (136.8)	1.06±0.06 (117.5)	0.004	0.817	1.25±0.1 (138.4)	1.13±0.11 (125.8)	0.88±0.06 (98.1)	0.003	0.128
Vitamin B2 (mg)	1.2±0.06 (119.7)	1.12±0.08 (112)	0.92±0.06 (92.4)	0.003	0.681	1.35±0.15 (136.4)	0.96±0.08 (96.6)	0.93±0.07 (92.2)	0.004	0.065
Niacin (mg)	15.93±0.64 (113.8)	15.23±0.96 (108.8)	11.9±0.54 (85)	<.0001	0.225	15.18±1.33 (110)	13.29±1.2 (95.1)	11±0.61 (78.1)	0.005	0.315

Vitamin B6 (mg)	2±0.09	1.82±0.13	1.47±0.07	<.0001	0.104	1.74±0.15	1.65±0.11	1.27±0.06	0.003	0.050
	(124.8)	(113.5)	(92.2)			(111.4)	(103.5)	(78.4)		
Vitamin B12 (μg)	4.03±0.3	3.58±0.62	3.34±0.65	0.342	0.851	3.71±0.73	3.6±0.63	3.62±0.76	0.933	0.705
	(167)	(149.5)	(141.1)			(164.8)	(151.4)	(147.7)		
Zinc (mg)	11.53±0.53	10.51±0.71	9.66±0.59	0.025	0.369	10.54±0.66	10.32±1.53	8±0.38	0.002	0.429
	(95.9)	(87.6)	(80.8)			(89.4)	(86.2)	(66.2)		
Iron (mg)	15.14±0.89	14.07±0.95	11.62±0.66	0.002	0.623	14.86±1.07	12.98±1.24	11.13±0.65	0.004	0.238
	(151.2)	(140.7)	(116.7)			(150.3)	(130)	(110.8)		
Sodium (mg)	2846.12±171.26	2616.02±240.68	2397.31±129.6	0.042	0.963	2154.03±192.34	2899.85±387.44	2379.94±169.71	0.382	0.048
	(118.1)	(109.1)	(101)			(87.8)	(120.6)	(99.8)		
Potassium (mg)	3024.83±138.78	2704.26±180.05	2098.48±107.19	<.0001	0.019	2522.13±169.4	2388.69±138.12	1851.31±112.56	0.001	0.107
	(116.3)	(104)	(80.8)			(99.5)	(92.2)	(70.4)		
Phosphorus (mg)	1124.19±48.41	1014.75±60.23	875.34±41.09	<.001	0.567	1042.2±54.9	875.67±49.97	824.21±44.64	0.002	0.552
	(140.4)	(126.9)	(109.6)			(132.9)	(109.8)	(102.2)		
Calcium (mg)	588.45±46.4	588.13±51.09	454.36±33.62	0.020	0.951	641.76±51.71	470.4±38.86	475.68±38.93	0.008	0.207
	(58.8)	(58.8)	(45.5)			(66)	(47.3)	(47)		
Magnesium (mg)	343.36±16.91	336.85±25.91	243.81±12.12	<.0001	0.161	287.53±17.78	255.94±11.13	228.98±12.16	0.009	0.464
	(111.9)	(109.9)	(79.8)			(98.9)	(85.7)	(75.4)		
SFA (mg)	17317.99±1159.55	13722.46±917.49	11226.01±810.64	<.0001	0.071	15603.79±1839.08	13347.26±1530.56	11536.41±863.58	0.040	0.573
	(30.3)	(27.9)	(29.6)			(31.5)	(29.3)	(29.2)		
MUFA (mg)	21407.53±1216.22	18939.03±1567.84	14631.32±1134.29	<.001	0.654	18700.9±1930.57	16845.2±2082.14	14027.84±911.88	0.029	0.902
	(37.3)	(37.4)	(37.1)			(37)	(36.5)	(35.6)		
PUFA (mg)	17825.48±1048.75	16436.67±1211.14	12011.48±955.1	<.001	0.203	15731.37±1861.71	13683.12±1512.18	12981.71±787.5	0.167	0.450
	(31.5)	(33.6)	(32.3)			(30.7)	(32.5)	(34.1)		
Cholesterol (mg)	240.03±18.67	193.54±21.85	180.69±21.68	0.036	0.598	271.54±52.22	174.35±30.89	157.72±15.97	0.035	0.098
	(79.9)	(64.5)	(60.4)			(90)	(58.1)	(52.7)		
Dietary fiber (g)	21.46±1.03	20.21±1.35	14.36±0.96	<.0001	0.033	18.42±1.32	15.78±0.78	12.8±0.84	0.001	0.040
	(98.9)	(93.2)	(66.5)			(89.9)	(75.4)	(60.3)		

[a] Data are age-adjusted Mean±SEM (% RDA). The RDAs are the current dietary reference intakes (DRIs, the 8th edition) established for Taiwanese people.

[b] The trend test was performed by using the general linear model to test whether the mean of variable has an ordered relationship across frailty level groups after adjusting for age (Model 1) or for age and energy intake (Model 2).

Table S5: Total energy and nutrients intakes per kilogram of body weight for the older men by frailty levels in 2014-2017 NAHSIT [a]

	65-74 year-old men					> 74 year-old men				
	**Robust**	**Prefrail**	**Frail**	***P* for trend [b]**		**Robust**	**Prefrail**	**Frail**	***P* for trend [b]**	
	**(n=230)**	**(n=150)**	**(n=12)**	***Model 1***	***Model 2***	**(n=86)**	**(n=114)**	**(n=24)**	***Model 1***	***Model 2***
Energy (Kcal/kg)	31.16±0.83 (95.4)	29.8±1.04 (91.7)	28.26±2.23 (87.9)	0.218	0.841	33.62±1.82 (103.4)	31.78±1.95 (96.9)	28.79±2.5 (85.3)	0.150	0.508
Carbohydrate (g/kg)	4.15±0.13 (188.9)	4±0.14 (183.3)	4.15±0.27 (191.3)	0.990	0.310	4.4±0.19 (197.9)	4.27±0.23 (190.1)	3.79±0.33 (163.6)	0.145	0.271
Protein (g/kg)	1.3±0.04 (136.1)	1.23±0.05 (129.9)	0.92±0.15 (98.3)	0.014	0.179	1.43±0.09 (138.3)	1.29±0.1 (125)	1.17±0.12 (111.9)	0.131	0.379
Fat (g/kg)	1.03±0.04 (94.4)	0.97±0.06 (89.2)	0.92±0.17 (85.1)	0.537	0.968	1.1±0.12 (101.7)	1.04±0.1 (95.5)	1±0.12 (89.7)	0.540	0.395
Vitamin A (μg/Kg RE)	18.09±1.58 (176.6)	15.62±1.94 (156.6)	18.34±4.62 (192.5)	0.960	0.719	16.13±2.03 (157.4)	16.41±1.92 (158.4)	19.93±4.33 (187.4)	0.455	0.385
Vitamin C (mg/Kg)	2.8±0.15 (165)	2.63±0.27 (157.2)	0.99±0.38 (66.2)	<.0001	0.001	3.17±0.38 (182.1)	2.74±0.3 (159.1)	1.92±0.52 (117)	0.028	0.032
Vitamin D (μg/Kg)	0.1067±0.0095 (62.7)	0.1105±0.0115 (66.1)	0.125±0.0851 (77.6)	0.831	0.759	0.1754±0.0265 (96.4)	0.1334±0.0237 (78.3)	0.132±0.0647 (94.8)	0.535	0.716
Vitamin E (mg/Kg α-TE)	0.1531±0.0079 (75)	0.1197±0.0089 (60)	0.1151±0.038 (60.8)	0.333	0.579	0.1671±0.0148 (78.8)	0.1292±0.0114 (62.7)	0.1337±0.0195 (71.1)	0.193	0.274
Vitamin B1 (mg/Kg)	0.0224±0.0009 (109.4)	0.0216±0.0012 (108.3)	0.0162±0.0033 (87)	0.073	0.315	0.0235±0.0014 (116.3)	0.0244±0.0024 (117.4)	0.0227±0.0028 (100.4)	0.800	0.398
Vitamin B2 (mg/Kg)	0.0203±0.0009 (91.9)	0.0184±0.0012 (84.7)	0.0189±0.0031 (90.1)	0.675	0.948	0.0217±0.0017 (96.5)	0.0196±0.0018 (87.5)	0.02±0.0035 (90.7)	0.671	0.943
Niacin (mg/Kg)	0.3072±0.015 (113.6)	0.2853±0.0171 (106.3)	0.2609±0.0463 (98.6)	0.335	0.738	0.3012±0.0221 (109.4)	0.3173±0.0297 (115)	0.2293±0.0273 (82.6)	0.060	0.107

Vitamin B6 (mg/Kg)	0.0355±0.0014 (130.8)	0.031±0.002 (115.8)	0.0264±0.0081 (102.5)	0.267	0.520	0.039±0.0026 (139.7)	0.0317±0.0023 (115.1)	0.0263±0.0031 (100.9)	0.006	0.005
Vitamin B12 (μg/Kg)	0.0787±0.01 (194.4)	0.0794±0.0137 (196.5)	0.1042±0.0449 (260.2)	0.578	0.483	0.0743±0.0108 (181)	0.061±0.0087 (147.1)	0.0468±0.009 (108.3)	0.072	0.202
Zinc (mg/kg)	0.2±0.01 (79.1)	0.19±0.01 (75.8)	0.15±0.02 (61.2)	0.034	0.292	0.23±0.02 (90.1)	0.19±0.01 (74.5)	0.17±0.02 (63.2)	0.029	0.021
Iron (mg/kg)	0.25±0.01 (147.9)	0.21±0.01 (126.9)	0.2±0.02 (122.4)	0.068	0.322	0.27±0.02 (157.7)	0.23±0.01 (133)	0.24±0.04 (131.1)	0.485	0.853
Sodium (mg/kg)	48.59±1.85 (120.5)	49.88±3.66 (123.2)	34.48±5.08 (83.6)	0.007	0.096	57.44±5.84 (137.4)	52.23±3.97 (126.5)	41.66±9.01 (105.3)	0.162	0.271
Potassium (mg/kg)	48.91±2.08 (84.8)	40.93±2.1 (72.1)	34.61±6.61 (63.7)	0.040	0.206	52.53±3.82 (89)	43.06±2.83 (73.6)	41.27±7.43 (72.5)	0.226	0.303
Phosphorus (mg/kg)	19.31±0.64 (142.7)	17.77±0.74 (132.6)	13.66±1.72 (104.5)	0.002	0.090	21.84±2.08 (157.9)	18.91±1.38 (137.2)	17.54±2.13 (128.5)	0.192	0.430
Calcium (mg/kg)	9.99±0.63 (58.9)	7.96±0.66 (47.7)	6.04±0.79 (38.1)	<.001	0.009	10.61±1.05 (61.2)	9.55±0.97 (55.5)	10.28±2.6 (60.7)	0.913	0.755
Magnesium (mg/kg)	5.72±0.24 (94.8)	4.5±0.24 (75.4)	3.46±0.42 (60.4)	<.0001	0.007	6.5±0.7 (108.4)	4.94±0.3 (81.8)	5.22±0.86 (84.2)	0.266	0.390
SFA (mg/kg)	291.28±11.9 (28.6)	301.08±16.15 (31.7)	384.11±110.51 (38.8)	0.405	0.182	317.96±32.85 (29.3)	348.03±32.57 (33.7)	311.26±36.63 (32.1)	0.893	0.151
MUFA (mg/kg)	381.9±16.65 (37)	364.3±21 (37.3)	292.41±41.9 (33.4)	0.051	0.230	418.35±50.76 (37)	399.09±41.26 (37.4)	389.38±50.94 (38.5)	0.692	0.290
PUFA (mg/kg)	340.16±15.92 (33.2)	294.9±22.9 (30.2)	237.47±38.39 (27.2)	0.013	0.165	361.67±40.09 (33.1)	288.98±32.73 (28)	289.48±50.45 (28.5)	0.282	0.829
Cholesterol (mg/kg)	3.85±0.19 (76.4)	4.35±0.32 (86.1)	2.46±0.87 (47.7)	0.122	0.225	3.95±0.36 (75.9)	4.09±0.43 (79.1)	3.46±0.62 (68.4)	0.518	0.951
Dietary fiber (g/kg)	0.34±0.02 (74.6)	0.25±0.02 (54.8)	0.22±0.03 (51.2)	<.001	0.018	0.36±0.03 (76.8)	0.29±0.02 (61.6)	0.29±0.04 (62.2)	0.204	0.305

[a] Data are age-adjusted Mean±SEM (% RDA). The RDAs are the current dietary reference intakes (DRIs, the 8th edition) established for Taiwanese people.

[b] The trend test was performed by using the general linear model to test whether the mean of variable has an ordered relationship across frailty level groups after adjusting for age (Model 1) or for age and energy intake (Model 2).

Table S6: Total energy and nutrients intakes per kilogram of body weight for the older men by stages of cognitive ability in 2014-2017 NAHSIT [a]

	**65-74 year-old men**					**> 74 year-old men**				
	**Normal cognition (n=266)**	**Mild cognitive impairment (n=156)**	**Cognitive impairment (n=51)**	***P* for trend [b]**		**Normal cognition (n=100)**	**Mild cognitive impairment (n=96)**	**Cognitive impairment (n=86)**	***P* for trend [b]**	
				Model 1	***Model 2***				***Model 1***	***Model 2***
Energy (Kcal/kg)	31±0.8 (100.2)	28.34±1.14 (92.9)	28.82±2.68 (80.8)	0.472	0.767	33.7±2.07 (93.5)	32.15±1.79 (79.5)	27.48±1.44 (75.6)	0.020	0.072
Carbohydrate (g/kg)	4.16±0.13 (169.5)	3.8±0.18 (156.7)	3.92±0.26 (143.3)	0.434	0.907	4.49±0.22 (157.7)	4.39±0.2 (128.6)	3.7±0.16 (117.9)	0.005	0.012
Protein (g/kg)	1.28±0.04 (133.2)	1.16±0.05 (124.1)	1.19±0.16 (97.8)	0.579	0.983	1.44±0.11 (115.2)	1.29±0.07 (96.1)	1.09±0.08 (89.1)	0.018	0.052
Fat (g/kg)	1.01±0.04 (95.8)	0.93±0.05 (86.2)	0.9±0.15 (70.6)	0.487	0.790	1.09±0.11 (80)	1.01±0.11 (76.3)	0.92±0.08 (78.3)	0.239	0.489
Vitamin A (μg/Kg RE)	17.4±1.44 (210.9)	16.27±2.02 (231.1)	12.29±3.33 (303)	0.180	0.172	17.19±1.77 (361.7)	17.73±1.65 (151.7)	14.52±1.79 (156.3)	0.271	0.406
Vitamin C (mg/Kg)	2.63±0.14 (186.8)	2.39±0.2 (151.5)	2.41±0.43 (87.7)	0.619	0.898	3.36±0.3 (143.4)	2.65±0.28 (107.4)	2.31±0.29 (115.2)	0.012	0.024
Vitamin D (μg/Kg)	0.1115±0.01 (55.5)	0.1107±0.012 (61.8)	0.0945±0.031 (39.6)	0.616	0.764	0.1797±0.0304 (47.9)	0.1315±0.0217 (45.1)	0.1181±0.0226 (38.6)	0.116	0.237
Vitamin E (mg/Kg α-TE)	0.1474±0.0065 (65.7)	0.1196±0.0065 (60.8)	0.1113±0.0162 (64.9)	0.056	0.016	0.1493±0.0108 (54)	0.1425±0.0123 (51.3)	0.1282±0.0109 (44.3)	0.150	0.495
Vitamin B1 (mg/Kg)	0.0226±0.001 (132.8)	0.0193±0.0011 (123.4)	0.0199±0.0025 (112.6)	0.384	0.537	0.0259±0.0023 (112.6)	0.0226±0.0011 (99)	0.0211±0.0016 (91.7)	0.093	0.362
Vitamin B2 (mg/Kg)	0.0204±0.0011 (106.9)	0.0174±0.0009 (106.4)	0.0156±0.0023 (109.4)	0.067	0.013	0.0199±0.001 (112)	0.0209±0.0014 (79)	0.0189±0.0023 (80.3)	0.690	0.728
Niacin (mg/Kg)	0.3058±0.014	0.2653±0.0174	0.267±0.0334	0.282	0.511	0.3393±0.0292	0.2776±0.0243	0.238±0.0213	0.007	0.013

	(104.5)	(97)	(62.5)			(97.1)	(74.4)	(74.4)		
Vitamin B6 (mg/Kg)	0.0348±0.0014	0.0307±0.0019	0.0292±0.004	0.197	0.320	0.0378±0.0031	0.0326±0.0016	0.0287±0.0021	0.025	0.042
	(110.7)	(106.6)	(76.8)			(100.9)	(79.9)	(73.5)		
Vitamin B12 (μg/Kg)	0.0829±0.013	0.0626±0.0059	0.0568±0.014	0.184	0.323	0.0744±0.0107	0.059±0.0069	0.0529±0.0091	0.113	0.308
	(138.2)	(145.6)	(71.9)			(181.9)	(134.3)	(76.6)		
Zinc (mg/kg)	0.2±0.01	0.18±0.01	0.19±0.02	0.671	0.449	0.21±0.01	0.21±0.01	0.17±0.01	0.019	0.125
	(86.4)	(79.3)	(59.2)			(82.5)	(62.6)	(55.3)		
Iron (mg/kg)	0.24±0.01	0.21±0.01	0.21±0.03	0.366	0.540	0.26±0.01	0.25±0.02	0.21±0.02	0.035	0.229
	(136.3)	(131.5)	(105.3)			(130.7)	(103.2)	(90.4)		
Sodium (mg/kg)	47.12±1.89	52.2±3.29	39.2±5.34	0.140	0.126	53.86±4.99	49.07±4.73	46.11±4.37	0.199	0.742
	(103.7)	(104.7)	(80.9)			(86.4)	(98.1)	(75.7)		
Potassium (mg/kg)	47.24±1.83	40.28±2.02	36.86±5.49	0.098	0.028	51.26±2.81	45.51±2.66	39.29±3.65	0.014	0.041
	(103.7)	(95.8)	(64.3)			(94.2)	(70.8)	(62)		
Phosphorus (mg/kg)	19.06±0.67	16.65±0.67	16.87±1.97	0.323	0.355	20.27±1.34	21.75±1.56	16.33±1.16	0.036	0.362
	(127.6)	(117.5)	(91.5)			(110.9)	(93.5)	(81.9)		
Calcium (mg/kg)	9.49±0.6	7.83±0.54	8.07±1.57	0.409	0.526	9.88±0.88	10.91±0.82	9.35±1.18	0.727	0.622
	(55.6)	(54.9)	(45.3)			(57)	(42)	(34.7)		
Magnesium (mg/kg)	5.56±0.23	4.4±0.21	4.06±0.55	0.023	0.001	5.81±0.28	6.05±0.58	4.49±0.39	0.006	0.173
	(104.8)	(90.8)	(64.1)			(89)	(73.9)	(58.6)		
SFA (mg/kg)	301.54±11.94	283.14±16.95	273.53±42.69	0.559	0.989	339.18±32.5	298.63±33.9	290.05±27.98	0.278	0.593
	(29.1)	(30.2)	(26.9)			(29.7)	(30.4)	(30.5)		
MUFA (mg/kg)	381.37±14.96	346.5±19.09	342.66±61.77	0.575	0.983	414.19±46.48	390.62±50.02	341.58±33.91	0.237	0.624
	(38)	(36.6)	(34.1)			(37.3)	(35.8)	(35.9)		
PUFA (mg/kg)	320.44±13.88	289.01±23.23	276±47.07	0.398	0.612	333.27±36.71	313.34±38.68	280.4±28.88	0.286	0.518
	(32)	(32.4)	(37.1)			(32.1)	(32.6)	(32.8)		
Cholesterol (mg/kg)	3.87±0.22	4.25±0.32	3.63±0.49	0.621	0.990	3.96±0.4	3.75±0.34	3.32±0.36	0.255	0.767
	(64.8)	(77.2)	(47.1)			(59.8)	(47.1)	(56.9)		
Dietary fiber (g/kg)	0.32±0.02	0.25±0.01	0.24±0.04	0.040	0.006	0.37±0.02	0.33±0.03	0.22±0.02	<.0001	<.0001
	(92.6)	(81.1)	(49.6)			(75.6)	(61.7)	(57.2)		

[a] Data are age-adjusted Mean±SEM (% RDA). The RDAs are the current dietary reference intakes (DRIs, the 8th edition) established for Taiwanese people.

[b] The trend test was performed by using the general linear model to test whether the mean of variable has an ordered relationship across frailty level groups after adjusting for age (Model 1) or for age and energy intake (Model 2).

Table S7: Total energy and nutrients intakes per kilogram of body weight for the older women by frailty levels in 2014-2017 NAHSIT [a]

	65-74 year-old women					> 74 year-old women				
	**Robust**	**Prefrail**	**Frail**	***P* for trend [b]**		**Robust**	**Prefrail**	**Frail**	***P* for trend [b]**	
	**(n=224)**	**(n=123)**	**(n=10)**	***Model 1***	***Model 2***	**(n=59)**	**(n=103)**	**(n=24)**	***Model 1***	***Model 2***
Energy (Kcal/Kg)	30.62±1.05	28.4±1.18	24.72±2.12	0.014	0.109	27.75±1.53	23.82±1.3	23.39±3.44	0.248	0.825
	(95.1)	(87)	(88.6)			(103.3)	(97.9)	(83.6)		
Carbohydrate (g/Kg)	4.27±0.12	3.98±0.16	3.74±0.26	0.067	0.992	4.03±0.23	3.34±0.18	3.24±0.47	0.132	0.263
	(189.8)	(173.4)	(179.3)			(201.6)	(195.2)	(164.6)		
Protein (g/Kg)	1.29±0.05	1.21±0.08	0.96±0.14	0.025	0.135	1.14±0.07	0.96±0.06	0.93±0.12	0.119	0.661
	(135.2)	(121.2)	(124.1)			(139.4)	(124.4)	(105.5)		
Fat (g/Kg)	0.98±0.05	0.88±0.07	0.7±0.11	0.022	0.186	0.8±0.07	0.76±0.06	0.78±0.17	0.892	0.154
	(93.5)	(85.2)	(82.5)			(100.3)	(92.4)	(84.1)		
Vitamin A (μg/Kg RE)	20.48±1.76	22.51±3.91	29.24±19.58	0.657	0.545	33.85±13.98	14.93±2.41	21.19±4.82	0.325	0.478
	(169.4)	(163.5)	(127)			(167)	(170.9)	(139.8)		
Vitamin C (mg/Kg)	3.61±0.26	2.97±0.28	1.85±0.24	<.0001	<.001	2.83±0.34	2.14±0.23	2.4±0.78	0.632	0.701
	(154.7)	(143.3)	(146)			(195.4)	(153.2)	(133.9)		
Vitamin D (μg/Kg)	0.1083±0.0113	0.1195±0.0154	0.0729±0.0187	0.152	0.446	0.0909±0.0179	0.0897±0.0133	0.0887±0.0262	0.943	0.575
	(65.4)	(66.3)	(57.6)			(102.1)	(77.5)	(69.9)		
Vitamin E (mg/Kg α-TE)	0.1535±0.0093	0.1412±0.0105	0.1485±0.0288	0.873	0.492	0.1265±0.0155	0.1228±0.0112	0.1137±0.0214	0.639	0.583
	(72.3)	(59.8)	(56.7)			(71.7)	(69.1)	(62.3)		
Vitamin B1 (mg/Kg)	0.0232±0.0012	0.0217±0.0015	0.0205±0.0028	0.371	0.855	0.0203±0.0018	0.0178±0.0013	0.0164±0.0038	0.357	0.739
	(110.6)	(96.7)	(101.1)			(126.2)	(108.5)	(101.2)		
Vitamin B2 (mg/Kg)	0.0208±0.0011	0.0207±0.0017	0.0214±0.0041	0.890	0.370	0.0222±0.0024	0.0158±0.0011	0.0166±0.0026	0.112	0.321
	(92.2)	(80.4)	(73.4)			(88.6)	(93)	(84.2)		
Niacin (mg/Kg)	0.2844±0.0112	0.2641±0.0149	0.1718±0.0179	<.0001	<.001	0.2731±0.0281	0.2085±0.0173	0.2055±0.0271	0.154	0.561

	(113.3)	(98.6)	(99.2)			(123.3)	(100.4)	(86.1)		
Vitamin B6 (mg/Kg)	0.0344±0.0017	0.0332±0.0024	0.0242±0.0026	0.001	0.170	0.0318±0.003	0.0255±0.0016	0.0247±0.0042	0.157	0.375
	(128.8)	(114.3)	(109.5)			(137.1)	(118)	(104.1)		
Vitamin B12 (μg/Kg)	0.0649±0.0055	0.0675±0.0074	0.0296±0.0073	0.001	0.009	0.0866±0.0192	0.0644±0.0148	0.0384±0.008	0.015	0.021
	(204.3)	(155.5)	(142.6)			(180.6)	(142)	(127.2)		
Zinc (mg/Kg)	0.2±0.01	0.19±0.01	0.14±0.02	0.015	0.085	0.19±0.02	0.15±0.01	0.14±0.02	0.049	0.102
	(78)	(70.6)	(74.5)			(83.3)	(81.1)	(65.7)		
Iron (mg/Kg)	0.27±0.01	0.26±0.03	0.2±0.03	0.069	0.552	0.25±0.02	0.21±0.01	0.2±0.03	0.200	0.431
	(142.9)	(124.2)	(126.5)			(153)	(145.4)	(120.3)		
Sodium (mg/Kg)	48.25±2.78	49.02±3.01	38.93±4.64	0.098	0.622	41.23±2.94	47.08±5.93	36.94±6.93	0.586	0.817
	(117.5)	(128.3)	(94.6)			(129.9)	(118.7)	(111.6)		
Potassium (mg/Kg)	52.39±2.43	48.5±3.81	32.6±5.82	0.002	0.062	48.17±2.97	36.76±2.31	34.21±6.51	0.056	0.084
	(81.9)	(70.8)	(65.6)			(87.6)	(77.6)	(66.9)		
Phosphorus (mg/Kg)	19.83±0.83	18.3±1.13	14.39±1.33	0.001	0.038	17.56±1.04	14.94±0.87	13.54±1.99	0.071	0.190
	(141.1)	(123.9)	(126)			(147.6)	(157.4)	(118)		
Calcium (mg/Kg)	10.8±0.84	10.7±1.42	8.86±2.14	0.403	0.963	11.13±0.88	8.38±0.84	7.57±1.74	0.070	0.167
	(55.9)	(46.9)	(48.9)			(57.3)	(63.3)	(54.2)		
Magnesium (mg/Kg)	6.25±0.31	5.44±0.42	3.94±0.52	<.001	0.021	5.22±0.31	4.42±0.32	3.8±0.65	0.057	0.101
	(92)	(73.8)	(68.9)			(97.1)	(99.7)	(73.9)		
SFA (mg/Kg)	279.44±16.29	258.51±18.4	188.48±29.76	0.009	0.121	238.85±21.31	229.82±18.43	227.13±58.06	0.850	0.345
	(29.9)	(30.6)	(30.5)			(31.4)	(30.1)	(32.2)		
MUFA (mg/Kg)	375.69±21	322.09±22.25	236.24±40.4	0.003	0.005	295.94±23.63	281.21±25.87	285.49±65.49	0.881	0.173
	(37.1)	(37.5)	(37.7)			(37.2)	(37.7)	(36.9)		
PUFA (mg/Kg)	312.63±19.43	289.19±30.41	266.15±48.38	0.381	0.554	261.77±25.22	243.99±21.23	258.63±52.14	0.958	0.160
	(31.9)	(31)	(30.9)			(30.7)	(31.4)	(30)		
Cholesterol (mg/Kg)	3.78±0.31	4.5±0.5	2.76±1.12	0.380	0.807	3.54±0.59	2.82±0.36	3.54±0.72	0.996	0.349
	(77)	(83.8)	(70.9)			(76.2)	(72.6)	(64.3)		
Dietary fiber (g/Kg)	0.39±0.02	0.34±0.03	0.21±0.04	<.001	0.006	0.31±0.02	0.26±0.02	0.27±0.04	0.445	0.915
	(70.6)	(55.4)	(53.1)			(79.2)	(70.6)	(47)		

[a] Data are age-adjusted Mean±SEM (% RDA). The RDAs are the current dietary reference intakes (DRIs, the 8th edition) established for Taiwanese people.

[b] The trend test was performed by using the general linear model to test whether the mean of variable has an ordered relationship across frailty level groups after adjusting for age (Model 1) or for age and energy intake (Model 2).

Table S8: Total energy and nutrients intakes per kilogram of body weight for the older women by stages of cognitive ability in 2014-2017 NAHSIT [a]

	**65-74 year-old women**					**> 74 year-old women**				
	**Normal cognition (n=189)**	**Mild cognitive impairment (n=152)**	**Cognitive impairment (n=105)**	***P* for trend [b]**		**Normal cognition (n=36)**	**Mild cognitive impairment (n=78)**	**Cognitive impairment (n=151)**	***P* for trend [b]**	
				Model 1	***Model 2***				***Model 1***	***Model 2***
Energy (Kcal/Kg)	31.19±1.04 (102.1)	27.51±1.66 (90.1)	25.22±1.12 (82.8)	<.001	0.788	29.33±1.91 (99.2)	26.75±1.45 (89.5)	22.52±1.22 (74.4)	0.007	0.196
Carbohydrate (g/Kg)	4.34±0.12 (172.1)	3.78±0.21 (149.5)	3.75±0.17 (146.9)	0.006	0.593	3.99±0.25 (157.1)	3.68±0.2 (142.2)	3.26±0.21 (123.7)	0.047	0.513
Protein (g/Kg)	1.32±0.06 (136)	1.2±0.08 (123.3)	1.02±0.07 (104.8)	0.001	0.519	1.25±0.07 (127.5)	1.12±0.1 (112.4)	0.91±0.04 (89.8)	<.001	0.374
Fat (g/Kg)	1±0.05 (98.4)	0.89±0.07 (87.4)	0.67±0.06 (66.9)	<.0001	0.231	0.91±0.08 (91.3)	0.87±0.08 (87.1)	0.66±0.05 (66.4)	0.018	0.972
Vitamin A (μg/Kg RE)	21.64±2.16 (222.5)	21.07±2.62 (216.7)	17.03±2.77 (174.4)	0.167	0.908	33.76±16.06 (361)	18.23±1.51 (187.4)	15.9±1.95 (148.1)	0.264	0.365
Vitamin C (mg/Kg)	3.63±0.25 (187.4)	3.21±0.31 (165.3)	2.21±0.28 (111.2)	<.001	0.028	2.72±0.33 (137.9)	2.59±0.29 (129.8)	1.96±0.2 (97.2)	0.051	0.122
Vitamin D (μg/Kg)	0.1114±0.0119 (57)	0.1075±0.0164 (55.3)	0.0963±0.0196 (49.8)	0.560	0.725	0.0801±0.0138 (43.7)	0.1225±0.0236 (62)	0.0812±0.0094 (38.9)	0.947	0.131
Vitamin E (mg/Kg α-TE)	0.1569±0.0089 (66.9)	0.14±0.0104 (60)	0.1123±0.0113 (48.4)	0.004	0.279	0.1308±0.0148 (56.7)	0.1363±0.013 (57.2)	0.1139±0.0081 (46.4)	0.324	0.483
Vitamin B1 (mg/Kg)	0.0235±0.0012 (134.8)	0.0221±0.0016 (125.9)	0.0189±0.0012 (106.6)	0.005	0.953	0.0225±0.0017 (125.1)	0.0215±0.0018 (119.4)	0.0158±0.0012 (88)	0.004	0.235
Vitamin B2 (mg/Kg)	0.0215±0.0013 (110.4)	0.0204±0.0017 (104.7)	0.0161±0.0014 (83)	0.006	0.594	0.0237±0.0025 (120.1)	0.018±0.0014 (90.5)	0.016±0.001 (79.2)	0.003	0.078

Niacin (mg/Kg)	0.2862±0.0123 (105.2)	0.2733±0.0196 (100.3)	0.2061±0.0106 (75.2)	<.0001	0.089	0.2934±0.0304 (106)	0.2535±0.0223 (90.8)	0.1981±0.0109 (70.2)	0.004	0.209
Vitamin B6 (mg/Kg)	0.0359±0.0017 (115.6)	0.0321±0.0025 (103.2)	0.0258±0.0016 (82.3)	<.0001	0.053	0.0314±0.0022 (100.3)	0.0322±0.0022 (101.1)	0.0231±0.0015 (71)	0.004	0.247
Vitamin B12 (μg/Kg)	0.0719±0.0052 (153.1)	0.0614±0.0113 (131.1)	0.0564±0.0144 (122.3)	0.324	0.994	0.0674±0.0145 (145.8)	0.0668±0.0138 (140.3)	0.056±0.012 (114.1)	0.555	0.972
Zinc (mg/Kg)	0.2±0.01 (87.3)	0.19±0.01 (80.3)	0.17±0.01 (72.1)	0.017	0.608	0.2±0.01 (84.7)	0.19±0.02 (80.4)	0.14±0.01 (58)	0.001	0.128
Iron (mg/Kg)	0.27±0.02 (137.9)	0.25±0.02 (129)	0.21±0.02 (109)	0.025	0.915	0.29±0.03 (149.2)	0.22±0.01 (110.8)	0.2±0.01 (95.4)	0.004	0.161
Sodium (mg/Kg)	48.73±2.37 (104.3)	45.48±3.95 (97.3)	41.76±2.55 (89.3)	0.037	0.802	35.84±2.77 (78.3)	46.01±4.89 (96.7)	42.51±3.88 (86.8)	0.168	0.003
Potassium (mg/Kg)	53.66±2.66 (106.2)	48.74±3.66 (96.4)	37.16±2.65 (73)	<.0001	0.039	46.31±3.79 (91.9)	45.47±2.49 (88)	33.41±1.97 (62.9)	0.004	0.141
Phosphorus (mg/Kg)	20.03±0.9 (128.8)	18.28±1.2 (117.4)	15.46±0.95 (98.8)	0.001	0.500	19.42±1.12 (123.4)	16.72±0.99 (105)	14.32±0.79 (88.6)	0.001	0.238
Calcium (mg/Kg)	10.74±0.88 (55.2)	10.77±1.02 (55.4)	7.87±0.75 (40.2)	0.014	0.635	12.04±1.19 (62.6)	8.51±0.63 (43.1)	8.65±0.77 (42.1)	0.016	0.246
Magnesium (mg/Kg)	6.14±0.31 (103)	5.96±0.49 (99.8)	4.42±0.33 (73.6)	<.001	0.339	5.61±0.45 (96.3)	4.83±0.26 (81.2)	4.11±0.27 (67.2)	0.007	0.235
SFA (mg/Kg)	296.13±16.85 (30.1)	247.07±19.25 (28.1)	194.51±16.57 (29.7)	<.0001	0.075	270.29±24.37 (30)	265.71±24.87 (30.3)	193.99±15.65 (29.6)	0.014	0.700
MUFA (mg/Kg)	380.35±21.39 (37.5)	343.23±32.32 (37.7)	250.27±22.77 (36.4)	<.0001	0.383	341.43±32.59 (37.4)	326.54±32.51 (36.8)	238.35±18.1 (35.4)	0.009	0.597
PUFA (mg/Kg)	319.39±19.77 (31.5)	290.93±26.82 (33)	222.09±24.79 (32.9)	0.002	0.576	294.88±35.15 (31.7)	271.96±30.2 (31.8)	225.58±18.35 (33.8)	0.101	0.572
Cholesterol (mg/Kg)	4.37±0.37 (75)	3.53±0.39 (60.4)	2.9±0.35 (49.6)	0.003	0.144	4.73±0.9 (77.3)	3.17±0.48 (52.5)	2.75±0.26 (46.5)	0.033	0.200
Dietary fiber (g/Kg)	0.38±0.02	0.36±0.03	0.28±0.02	0.001	0.305	0.34±0.03	0.3±0.02	0.24±0.02	0.012	0.182

	(89.6)	(86.1)	(65)	(84.3)	(71)	(55.9)

[a] Data are age-adjusted Mean±SEM (% RDA). The RDAs are the current dietary reference intakes (DRIs, the 8th edition) established for Taiwanese people.

[b] The trend test was performed by using the general linear model to test whether the mean of variable has an ordered relationship across frailty level groups after adjusting for age (Model 1) or for age and energy intake (Model 2).
